# Data-Driven Approach to Derive Equation for Predicting Ultimate Shear Strength of Reinforced Concrete Beams Without Stirrups

**DOI:** 10.3390/ma18112446

**Published:** 2025-05-23

**Authors:** Menghay Phoeuk, Dong-Yeong Choi, Suchart Limkatanyu, Minho Kwon

**Affiliations:** 1 Department of Civil Engineering, Gyeongsang National University, Jinju 52828, Republic of Korea; menghayph@gnu.ac.kr (M.P.); ced5189@gnu.ac.kr (D.-Y.C.); 2 Department of Civil Engineering, Prince of Songkla University, Songkhla 90112, Thailand; suchart.l@psu.ac.th

**Keywords:** data-driven approach, shear strength, artificial neural networks, Bayesian optimization, synthetic data

## Abstract

Shear failure in reinforced concrete (RC) beams is abrupt and brittle, occurs without warning, and leaves no opportunity for internal stress redistribution. Despite the critical need for accurate shear strength assessment, existing methods vary widely across regions, leading to inconsistencies in practice. This study presents a unified shear strength equation for non-prestressed rectangular RC beams without stirrups, developed for simplicity and broad applicability. The model requires only basic geometric and material properties and applies to both shear-slender and non-shear-slender beams. It was formulated using a data-driven approach that combines an extensive experimental database collected up to 2007 with advanced computational techniques, including Artificial Neural Networks, Generative Adversarial Networks, and Bayesian optimization. The proposed equation was evaluated against established shear provisions, such as ACI 318-25 and CSA A23.3-24, and benchmarked with an experimental database. The results show that the model improves prediction accuracy, reduces uncertainty, and provides a more consistent method for shear strength assessment. The robustness of the equation was further confirmed through additional experimental database gathered after 2007, demonstrating strong agreement with test results and lower prediction uncertainty than current code provisions. These findings support the potential adoption of the proposed equation in engineering practice.

## 1. Introduction

Reinforced concrete (RC) beams are susceptible to sudden shear failures, which occur without warning and prevent internal stresses from being redistributed. The abrupt nature of these failures presents a significant challenge in structural engineering, as it directly affects the safety and stability of structures. The shear performance of RC beams is influenced by several interconnected factors, including concrete strength, shear-span-to-depth ratio, longitudinal reinforcement ratio, member depth, crack width and the presence of transverse reinforcement [1,2].

Over the years, numerous analytical and empirical models have been proposed to estimate the shear strength of RC beams. These include the strut-and-tie model, the modified compression field theory (MCFT), rotating and fixed-angle softened truss models, the critical shear crack theory (CSCT), mechanical models, and various design provisions [3,4,5]. Broadly, these approaches can be categorized as empirical, mechanics-based, or semi-empirical. Their accuracy has been assessed through benchmarking against experimental data, such as those reported for shear-slender (flexural) beams by Ahmad and Bhargava [3] and Ma et al. [4] and for non-shear-slender (deep) beams by Ismail et al. [5]. However, the highly nonlinear relationship between shear strength and influencing parameters—such as effective depth, concrete compressive strength, and reinforcement ratio—makes the determination of model coefficients complex. This complexity limits the direct use of these models in design codes. As a result, considerable variation persists among regional design methodologies, leading to inconsistencies in engineering practice [6,7].

In current practice, structural engineers classify RC beams as shear-slender or non-shear-slender based on their shear-span-to-depth ratio (a/d) before determining their shear capacity [6,7]. Beams with a/d greater than two are typically analyzed using semi-empirical equations, while beams with a/d less than two are evaluated using the strut-and-tie method (STM) [8]. These methods are influenced by independent research and assumptions that vary across regions. As a result, there is no unified method for predicting ultimate shear strength, which causes discrepancies in engineering practice and complicates the standardization of shear design.

This study proposes a unified shear strength equation for non-prestressed rectangular RC beams without stirrups. The model is designed for easy application, requiring only beam geometric properties and material strength as inputs while being applicable to both shear-slender and non-shear-slender beams. The proposed equation is derived through a data-driven method that combine an extensive experimental database with advanced computational techniques—including Artificial Neural Networks (ANNs), Generative Adversarial Networks (GANs), and Bayesian optimization (BO). The equation aims to provide a consistent, unified approach to predicting ultimate shear strength. Its performance is assessed by comparing it with existing shear strength provisions using a comprehensive experimental dataset as a benchmark.

## 2. Background Knowledge

### 2.1. Shear Behavior of RC Beams Without Stirrups

**Figure 1:**
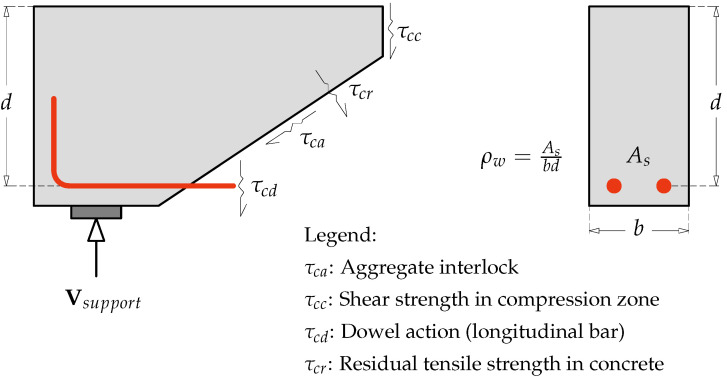
Transfer mechanisms of shear in a reinforced concrete beam without stirrups.

Regardless of the beam’s shear slenderness or depth, the shear resistance in RC beams without stirrups depends on several key factors. These include the uncracked concrete compression zone ($\tau_{cc}$), residual tensile strength across the crack ($\tau_{cr}$), aggregate interlock ($\tau_{ca}$), and the dowel action ($\tau_{cd}$) of the main longitudinal reinforcement [9,10,11], as illustrated in Figure 1.

The individual effects of these parameters on shear strength are difficult to separate due to their complex interactions. For instance, a higher longitudinal reinforcement ratio ($\rho_{w}$) enhances shear strength in RC beams by limiting the penetration of flexural cracks into the compression zone, thereby boosting shear capacity [12]. This also results in narrower cracks, which enhance aggregate interlock and further strengthen shear resistance [11,13]. Angelakos et al. [14] demonstrated that increasing $\rho_{w}$ from 0.5% to 2.09% increased the shear strength of RC beams by approximately 62%. Moreover, the compressive strength of concrete ($f_{c}^{\prime }$) plays a crucial role by improving its diagonal tensile capacity, thereby directly contributing to shear strength. However, this effect tends to level off when $f_{c}^{\prime }$ is above 90 MPa [15].

On the other hand, the shear slenderness of an RC beam, which is characterized by the shear-span-to-depth ratio (a/d), significantly influences its shear capacity. For example, RC beams with a/d ratios less than 2.5 can withstand considerably higher shear forces compared to those with a/d ratios greater than 2.5 that have the same amount of longitudinal reinforcement [2,10]. This disparity in shear capacity is due to different shear-transfer mechanisms: deeper beams (a/d<2.5) rely on arch action, which enhances shear resistance; while slender beams (a/d>2.5) predominantly rely on truss action for shear transfer [8,16]. Additionally, regarding the size effect, Sneed and Ramirez [17] confirmed that shear capacity of RC beams decreases as beam depth increases. They observed that beams with greater depth tend to develop wider cracks and exhibit reduced shear strength compared similarly reinforced beams of lesser depth. This size effect is acknowledged in shear provisions such those in ACI 318-25 [6] and CSA A23.3-24 [7].

### 2.2. Provisions for Shear Strength of RC Beams Without Stirrups

The current section presents the established standardized methods for determining the shear capacity of RC beams without stirrups according to design codes ACI 318-25 [6] and CSA A23.3-24 [7]. These design codes classify beams into different categories according to their geometric characteristics and loading conditions, which usually results in two main types: flexural beams and deep beams. Design codes apply the sectional method to calculate shear strength for flexural beams and use the strut-and-tie method (STM) when determining shear strength for deep beams [8].

#### 2.2.1. Calculation of RC Beams’ Shear Strength as per ACI 318-25

A beam is classified as a deep beam when its clear span is less than four times its total depth or when a concentrated load is applied within twice the member depth from the support. In such cases, the STM is used for shear strength evaluation. Beams that do not meet these criteria are classified as flexural beams, with shear strength determined using standard equations.

For flexural RC beams without shear reinforcement and zero axial force, ACI 318-25 [6] defines shear strength as

(1) $$V_{c,aci}=\min\left\{\begin{array}{c} 0.66\lambda_{d}{\left(\rho_{w}\right)}^{1/3}\sqrt{f_{c}^{\prime }}bd\\ 0.42\sqrt{f_{c}^{\prime }}bd \end{array}\right.\;\mathrm{where}\;\lambda_{d}=\sqrt{\frac{2}{1+0.004d}}\leq 1$$

For deep beams, shear strength is determined using the STM, which represents force transfer through compression struts, tension ties, and nodal zones and is expressed as

(2) $$V_{c,aci}=\min\left\{\begin{array}{c} 0.85\beta_{c}\beta_{s}f_{c}^{\prime }A_{cs}\sin\theta \\ A_{ts}f_{y}\sin\theta \\ 0.85\beta_{c}\beta_{n}f_{c}^{\prime }A_{nz}\sin\theta \end{array}\right.$$

where $A_{cs}$ is the cross-sectional area of the strut; $A_{nz}$ is the nodal zone area; $A_{ts}$ is the reinforcement area; and θ is the strut inclination angle. The factor $\beta_{c}$ accounts for strut and node confinement; $\beta_{s}$ represents the effectiveness of the strut in transferring the load; and $\beta_{n}$ modifies the effective concrete strength in the nodal zone.

#### 2.2.2. Calculation of RC Beams’ Shear Strength as per CSA A23.3-24

Similar to ACI 318-25 [6], CSA A23.3-24 [7] classifies a beam as a deep beam when its span-to-depth ratio is two or less. In such cases, the STM is recommended for shear strength evaluation. Beams that exceed this ratio are categorized as flexural beams, where shear strength is determined using sectional methods.

For flexural beams, CSA A23.3-24 [7] provides two procedures for shear capacity evaluation: the simplified method and the general method. This study adopts the simplified method, which defines shear strength as

(3) $$V_{c,csa}=\min\left\{\begin{array}{c} \beta \sqrt{f_{c}^{\prime }}bd\\ 0.25f_{c}^{\prime }bd \end{array}\right.\;\mathrm{where}\;\beta =\frac{230}{1000+s_{ze}}\;\mathrm{and}\;s_{ze}=\frac{35s_{z}}{15+a_{g}}\leq 0.85s_{z}$$

For deep beams, shear strength is determined using STM, which models force transfer through compression struts, tension ties, and nodal zones. It defines shear strength as

(4) $$V_{c,csa}=\min\left\{\begin{array}{c} \eta_{s}f_{c}^{\prime }A_{cs}\sin\theta \\ A_{ts}f_{y}\sin\theta \\ \eta_{n}f_{c}^{\prime }A_{nz}\sin\theta \end{array}\right.$$

where $A_{cs}$ is the cross-sectional area of the strut, $A_{nz}$ is the nodal zone area, $A_{ts}$ is the reinforcement area, and $\eta_{s}$ and $\eta_{n}$ are efficiency factors for the strut and nodal zone (in which $\eta_{s}=\frac{1}{0.8+170\epsilon_{1}}\leq 0.85\;\mathrm{with}\;\epsilon_{1}=\epsilon_{s}+\left(\epsilon_{s}+0.002\right)\cot^{2}\theta_{s}$).

### 2.3. Data-Driven Modeling for Shear Strength of RC Beams

Machine learning (ML) has been widely applied to predict the ultimate shear strength of RC structural elements, including beams [18,19], columns [20], and beam–column joints [21]. These data-driven models outperform traditional methods by providing higher accuracy and capturing complex structural behaviors that standard equations often do not consider. ML-based shear strength prediction relies on two key components: the dataset and the learning algorithm. Table 1 presents recent studies on data-driven modeling for predicting the ultimate shear strength of RC beams without stirrups, detailing dataset sizes, optimal algorithms, and key findings. For a broader exploration of ML applications in structural engineering, see the comprehensive review by Thai [22].

As shown in Table 1, ML models exhibit superior predictive performance compared to existing code provisions and mechanics-based models for estimating the shear strength of RC beams without stirrups. These models, developed by various researchers using diverse datasets, consistently demonstrate improved accuracy over traditional approaches. These collective findings highlight the potential of ML-based approaches to advance shear strength prediction and position data-driven modeling as a promising method for achieving more reliable and cost-effective design.

Nonetheless, the results reported in Table 1 also indicate that data-driven models can exhibit high coefficients of variation (COVs), especially when datasets contain beams with significantly differing shear-span-to-depth (a/d) ratios. For example, in the study by Prayoonwet et al. [19], the COVs for Datasets A and C were found to be 27.1% and 51.4%, respectively, due to the presence of both high and low a/d values. On the other hand, when Dataset B was limited to beams with a/d ratios less than 2.5, the COV decreased to 17.2%, which is a result similar to that reported by Megahed [23]. Additionally, for beams with a/d ratios greater than 2.4, Lee and Kang [18] reported a COV of 4.7%, while David et al. [24] found a COV of 14.0%. These results highlight improved consistency when models are applied to more homogeneous data subsets.

Data-driven models outperform traditional methods in predicting the shear strength of RC beams without stirrups, yet their performance declines when faced with wide variations in shear-span-to-depth (a/d) ratios, particularly for datasets exhibiting a mix of flexural beam and deep beam specimens. This points to the need for improved preprocessing strategies and model architectures that better capture structural diversity. There is also potential to advance ML frameworks capable of delivering consistent, reliable predictions beyond narrow, dataset-specific conditions.

### 2.4. Bayesian Optimization

Bayesian optimization (BO) functions as an effective method for optimizing costly black-box functions within noisy environments, which makes it suitable for hyperparameter tuning and structural design optimization [25]. BO develops a probabilistic surrogate model that frequently utilizes Gaussian Processes (GPs) to mimic the objective function and directs the search through an acquisition function toward promising areas, unlike grid or random search methods. The BO method achieves a balance between exploration and exploitation, which leads to fewer expensive function evaluations while simultaneously enhancing solution accuracy. The combination of BO with ML and physics-informed models has widened its application potential for engineering challenges [26].

### 2.5. Generative Adversarial Networks (GANs)

Training ML models or performing data-driven analyses demands extensive, high-quality datasets. However, acquiring such data is often costly, challenging, or restricted by privacy concerns. To overcome this limitation, Generative Adversarial Networks (GANs) have emerged as a powerful approach for synthesizing data that closely mirror actual distributions [27]. First introduced by Goodfellow et al. [28] in 2014, GANs were initially designed to enhance data privacy. Since then, they have evolved, giving rise to specialized models such as Tabular GAN (TGAN) by Xu and Veeramachaneni [29], tailored for generating tabular data.

GANs function through an adversarial framework involving two models: the generator (G), which learns the data distribution and creates synthetic samples, and the discriminator (D), which distinguishes real from generated data. This continuous feedback loop forces the generator to refine its outputs, making them increasingly realistic. Recognizing GANs’ potential in generating high-fidelity synthetic data, researchers have developed advanced variations such as the conditional GAN (CGAN) and CTGAN to further improve generative performance [30].

In civil engineering, data are often highly correlated. For example, the shear strength of an RC beam depends on both its geometric properties and material strength, as discussed in Section 2.1. While GANs can expand datasets by capturing distributions, maintaining essential relationships between variables remains challenging. Therefore, using synthetic data in structural engineering requires a strategic approach.

This study employs a GAN-based approach, using the Conditional Tabular GAN (CTGAN) to generate synthetic independent variables such as concrete strength and beam dimensions. CTGAN was chosen for its effectiveness in modeling tabular data that include both continuous and categorical features, which aligns well with the characteristics of structural engineering datasets. The dependent variable, ultimate shear strength, is then predicted using an ANN model trained on actual experimental data. This combined method allows the expansion of the dataset with realistic synthetic inputs while maintaining the fundamental relationships between RC beam’s properties and their ultimate shear strength through the ANN model.

## 3. Materials and Methods

### 3.1. Overview of the Study

**Figure 2:**
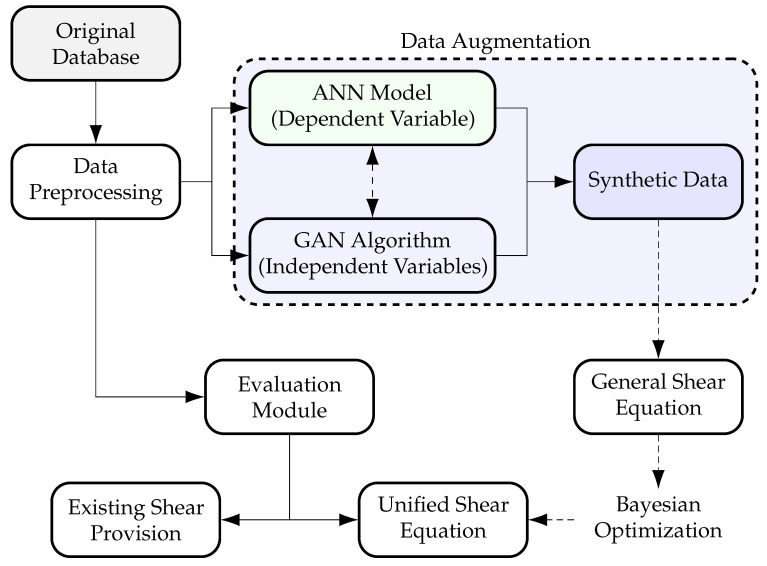
Flow chart providing an overview of this study.

This study aims to develop a unified shear equation for predicting the ultimate shear strength of non-prestressed rectangular RC beams without stirrups using a data-driven approach. As shown in Figure 2, the methodology begins with data compilation and preprocessing. An ANN model is trained on this dataset to establish a predictive framework for shear strength. To enhance data diversity, a GAN-based approach generates additional specimens by varying key independent variables such as material strength and beam geometry. These generated properties are then input into the trained ANN model to compute the corresponding shear strength, forming an expanded synthetic dataset.

The initial form of the shear equation is derived from configurational and constitutive parameters identified in the literature to ensure a foundation rooted in physical principles. This formulation undergoes refinement through a Bayesian optimization (BO) framework, which utilizes the expanded synthetic dataset. The synthetic data are used for model fitting, while the original dataset is reserved for validation. Finally, the derived equation is evaluated using the original dataset and compared with existing shear provisions to assess predictive accuracy and generalization capability.

### 3.2. Bibliographic Database

This study employs a database comprising 1849 experiments, compiled from sources in the literature over about 50 years (1948 to 2007). Originally compiled by Collins et al. [2], this database includes recorded test results of non-shear-reinforced RC beams subjected to flexural test configurations with both point load and distributed load protocols. This dataset has been used for evaluating the existing shear models [1,31] as well as for training and testing various ML models [18,19]. The database documents key properties such as beam geometry, concrete strength, longitudinal reinforcement, maximum aggregate size, shear-span-to-depth ratio (a/d), loading configurations, failure modes, and ultimate shear capacities.

For this study, the dataset was refined to align with the objectives. Specifically, we selected only beams that failed in shear and had an a/d ratio greater than 1, a rectangular section, zero axial force, and widths and/or heights exceeding 100 mm. After applying these criteria, the dataset was reduced to 1275 observations. Table 2 summarizes the key statistical characteristics of parameters within dataset, including their respective minimum, maximum and average values.

### 3.3. Development of ML Model: Model Configuration

This study employs an ANN to establish the relationship between nine input features and the ultimate shear strength (output) of RC beams, as detailed in Table 2. The theoretical and mathematical background of ANNs is not discussed here, as it is well established in the literature. For detailed explanations of ANNs applied to regression problems, the reader is referred to [32,33]. The model development process consists of two key stages:

i. Data normalization: input and output variables were standardized using normalization techniques to improve model convergence and predictive accuracy.
ii. Model optimization: neural network architecture and key hyperparameter were systematically fine-tuned through BO search guidance to achieve optimal performance.

Python served as the primary programming language for data analysis and model development. To enhance computational efficiency and streamline implementation, open-source libraries such as Pandas, TensorFlow, and Scikit-learn were employed.

#### 3.3.1. Data Normalization: Min–Max Scaling

Min–max scaling was applied to both input and output variables by transforming features into a fixed range of [0,1], using the following equation:

(5) $$x_{i,\mathrm{scaled}}=\frac{x_{i}-\min\left(x\right)}{\max(x)-\min(x)}$$

where $x_{i}$ represents the original data point, while min(x) and max(x) denote the minimum and maximum values in the dataset, respectively. This technique is effective for distance-based algorithms, such as k-nearest neighbors (KNN) and gradient-based optimization models, where inconsistent feature ranges can hinder learning efficiency [34]. By enforcing uniform feature scaling, min–max normalization prevents dominant features from skewing the learning process, which ensures stable and reliable model performance.

#### 3.3.2. Model Optimization: Bayesian Optimization (BO) Search Guidance

**Figure 3:**
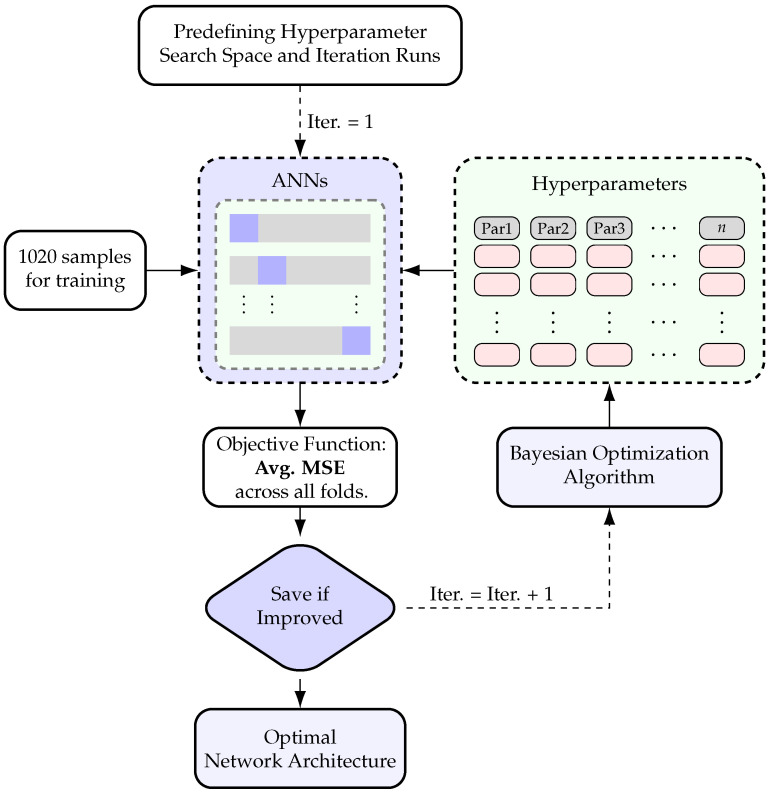
Bayesian–ANN framework for identifying the optimal network architecture and hyperparameters.

The dataset is randomly split into training and test sets at an 80:20 ratio, yielding 1020 instances for training and 255 instances for testing. The ANN model is trained using the 9 input features listed in Table 2 as model inputs and ultimate shear strength (target) as the model output. To refine the model, the training set undergoes further subdivision into internal training and testing subsets to explore different splits during network architecture and hyperparameter fine-tuning following the procedure illustrated in Figure 3.

As shown in Figure 3, BO guides the search for the optimal network architecture and hyperparameters. This process integrates five-fold cross-validation to ensure that the selected network architecture and hyperparameters perform well across different data splits. Once the optimal architecture and hyperparameters are identified, the model undergoes final training using the 1020-sample training set and is validated with the 255-sample testing set. In this context, BO aims to minimize the average cross-validation error, which serves as the criterion for tuning both the ANN architecture and key hyperparameters.

### 3.4. Generative Adversarial Network (GAN) Algorithm for Data Augmentation

The data augmentation process in this study consists of two main steps. First, the GAN algorithm generates independent variables, specifically the nine input features listed in Table 2. These generated features are then fed into the trained ANN model to compute their corresponding dependent variable (ultimate shear strength), forming the complete synthetic dataset.

**Figure 4:**
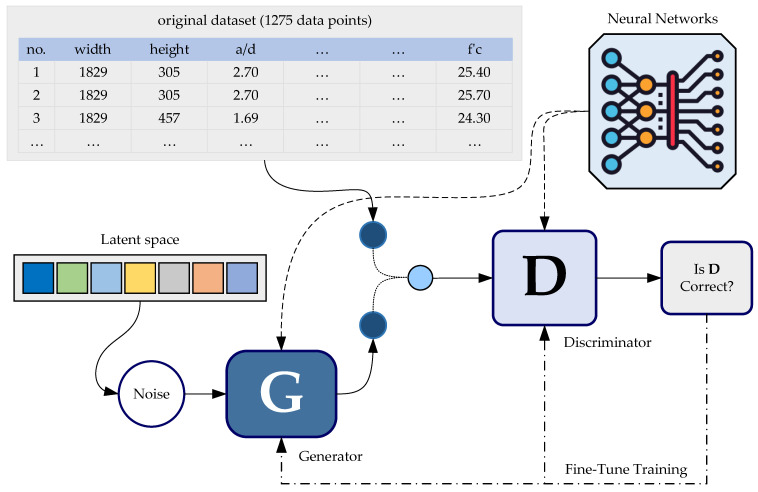
Flow of internal operations in generative adversarial networks (GANs).

As discussed in Section 2.5, GANs play a key role in generating high-fidelity synthetic data. They function through an iterative competition between a generator (G) and a discriminator (D). The generator G starts with random noise and refines its output based on feedback, while the discriminator D aims to distinguish real from synthetic data. Figure 4 illustrates this process. Over successive iterations, the generator G improves its ability to produce realistic data, while the discriminator D enhances its capacity to detect fakes. The process continues until the discriminator D can no longer reliably differentiate between real and synthetic data, indicating the generator’s proficiency [28,29]. For implementation, the Synthesizer feature in GAN-based deep learning techniques, developed by the Synthetic Data Vault [30], is used to generate synthetic data.

### 3.5. Optimization Framework for Deriving Shear Equation

#### 3.5.1. General Form of Shear Equation for RC Beams

Decades of experimental studies have established that the primary factors influencing shear resistance in RC beams without stirrups include the beam depth (*d*), longitudinal reinforcement ratio ($\rho_{w}$), concrete compressive strength ($f_{c}^{\prime }$), aggregate size ($a_{g}$) and shear-span-to-depth ratio (a/d) [2,11]. A statistical analysis of 784 experimental tests conducted by David et al. [24] further confirmed the dominant role of these variables in the shear behavior of RC beams without stirrups.

Building on these insights, the authors propose a shear strength model that integrates six key parameters: beam depth (to capture the size effect), dowel action of longitudinal reinforcement, aggregate size, shear-span-to-depth ratio, concrete strength, and effective cross-sectional area. These factors are incorporated into the general shear strength equation for RC beams without stirrups, as shown in Equation (6).

(6) $$V_{c}=\alpha_{1}\cdot {\left(\frac{\alpha_{2}}{d}\right)}^{\alpha_{3}}\cdot {\left(\rho_{w}\right)}^{\alpha_{4}}\cdot {\left(\frac{a_{g}}{\alpha_{5}}\right)}^{\alpha_{6}}\cdot {\left(\frac{1}{a/d}\right)}^{\alpha_{7}}\cdot {\left(f_{c}^{\prime }\right)}^{\alpha_{8}}\cdot bd$$

This equation is subject to the following constraints on individual terms to ensure physical relevance and model robustness:

$$\begin{array}{c} \alpha_{9}\leq {\left(\frac{\alpha_{2}}{d}\right)}^{\alpha_{3}}\leq \alpha_{10};\;\alpha_{11}\leq {\left(\rho_{w}\right)}^{\alpha_{4}}\leq \alpha_{12};\;\alpha_{13}\leq {\left(\frac{a_{g}}{\alpha_{5}}\right)}^{\alpha_{6}}\leq \alpha_{14};\\ \alpha_{15}\leq {\left(\frac{1}{a/d}\right)}^{\alpha_{7}}\leq \alpha_{16}\;\mathrm{and}\;\alpha_{17}\leq {\left(f_{c}^{\prime }\right)}^{\alpha_{8}}\leq \alpha_{18} \end{array}$$

#### 3.5.2. Bayesian Optimization (BO) Framework: Refining the Shear Equation

To derive the proposed shear strength equation for RC beams without stirrups, it is necessary to determine the parameter vector $\alpha_{i}$ (where i=1,2,…,18) in Equation (6). This study employs BO to refine the equation by fitting it to a synthetic dataset to ensure improved accuracy and predictive capability.

**Figure 5:**
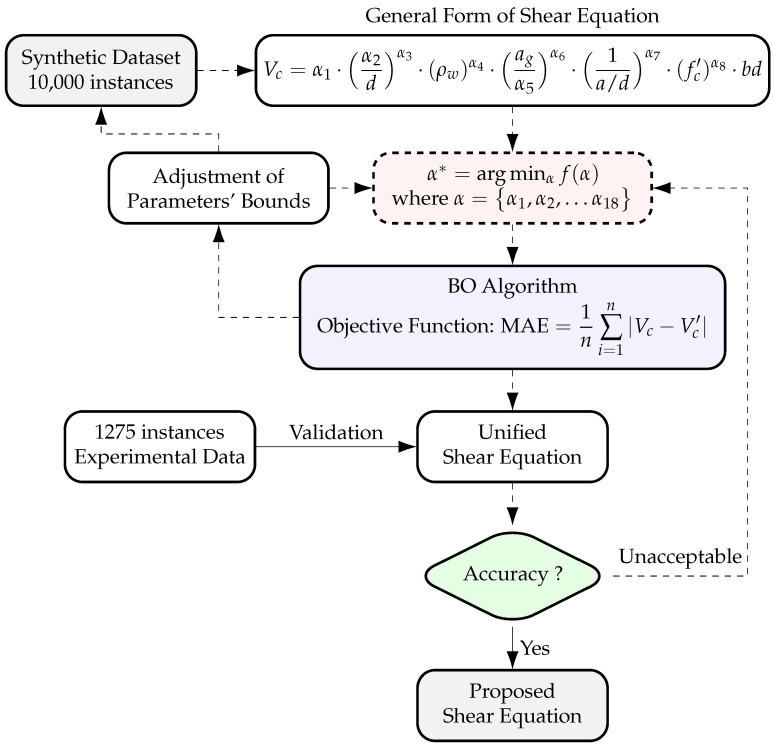
Bayesian optimization (BO) framework for refining the shear equation.

Figure 5 illustrates the BO framework used in this refinement process. The optimization begins with the generation of a synthetic dataset, which is then fed into Equation (6). The BO algorithm iteratively updates the parameter vector $\alpha_{i}$, searching for an optimal solution that maximizes the equation’s generalization performance.

As reflected in Equation (6), the shear stress, defined as $V_{c}/bd$, is governed by multiple power-law terms, including ${\left(\alpha_{2}/d\right)}^{\alpha_{3}}$, ${\left(\rho_{w}\right)}^{\alpha_{4}}$, ${\left(a_{g}/\alpha_{5}\right)}^{\alpha_{6}}$, ${\left(1/(a/d)\right)}^{\alpha_{7}}$, and ${\left(f_{c}^{\prime }\right)}^{\alpha_{8}}$. To enable efficient model calibration, lower and upper bounds are imposed on each term and embedded into the BO framework. During the optimization process, these parameters are iteratively updated to refine the equation and enhance predictive accuracy. The final outcome of the framework, as depicted in Figure 5, is the optimal set of parameter values $\alpha_{i}$ (where i=1,2,…,18). In this refinement, the open-source “skopt” library in Python is employed to implement the BO algorithm.

### 3.6. Evaluation Metrics

Four evaluation metrics are employed to evaluate the prediction efficiency of the ANN model in predicting the ultimate shear strength of RC beams. These metrics measure the accumulated error in predictions based on actual observations (experimental results). The statistical evaluation metrics used include the coefficient of determination ($R^{2}$), mean absolute error (MAE), root mean square error (RMSE), and mean absolute percentage error (MAPE). Equations (7)–(10) define the mathematical formulations of these metrics, where *n* represents the total number of test dataset records, while *y* and $y^{\prime }$ denote the measured and predicted shear strength of RC beams, respectively. Generally, $R^{2}$ values range from 0 to 1, with values closer to 1 indicating better model fitting. To assess modeling error, MAE, RMSE, and MAPE values are used, with smaller values indicating less error between prediction and measurement.

(7) $$R^{2}\left(y,y^{\prime }\right)=1-\frac{\sum {\left(y-y^{\prime }\right)}^{2}}{\sum {\left(y-\bar{y}\right)}^{2}}$$

(8) $$\mathrm{MAE}=\frac{1}{n}\sum \left|y-y^{\prime }\right|$$

(9) $$\mathrm{RMSE}=\sqrt{\frac{1}{n}\sum {\left(y-y^{\prime }\right)}^{2}}$$

(10) $$\mathrm{MAPE}=\frac{1}{n}\sum \left|\frac{y-y^{\prime }}{y}\right|$$

Additionally, the statistical ratio $V_{\exp}/V_{\mathrm{pred}}$ is employed to assess the conservatism of the proposed equation relative to existing provisions. A ratio closer to 1 indicates higher predictive accuracy, whereas a larger ratio reflects a more conservative estimate.

## 4. Results and Discussion

### 4.1. ANN Model for Predicting Shear Strength of RC Beams Without Stirrups

#### 4.1.1. Optimal Network Architecture and Hyperparameters for ANN Model

Following the procedure outlined in Figure 3, the search spaces for ANN architecture and key hyperparameters were first defined. These included the number of hidden layers; the number of neurons per layer; and key hyperparameters such as activation functions, optimizer, learning rate, batch size, and number of epochs. The number of iterations of the BO framework runs was initially set and increased as the search space was refined. This iteration was continued until no further performance gains were observed.

**Figure 6:**
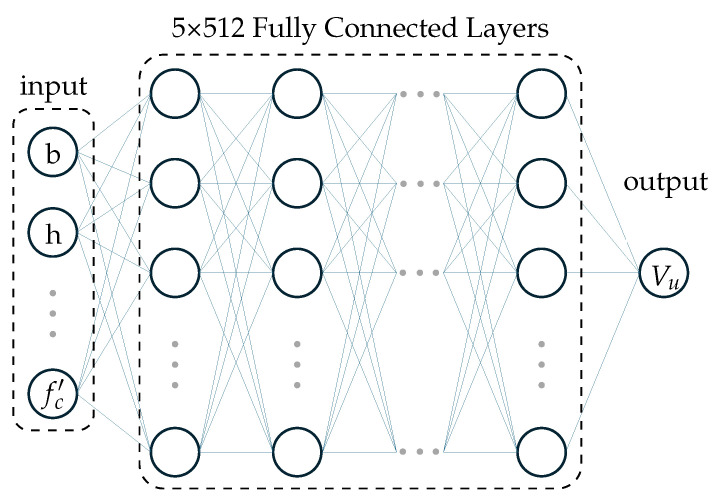
Schematic illustration of the optimal ANN architecture used in training.

The optimal architecture consisted of five hidden layers, each having 512 neurons. The activation function between the input and the first hidden layer as well as between the hidden layers was ReLU, while the output layer activation function was linear. The optimal hyperparameters, optimized using a BO framework incorporating five-fold cross-validation, were identified as follows: optimizer, Adam; learning rate, 0.0001; batch size, 64; and number of epochs, 50. This framework ensured that the ANN architecture and hyperparameters performed well overall across different data splits, resulting in a model with good generalization capability on new unseen data. A schematic illustration of the resulting optimal ANN architecture is presented in Figure 6.

#### 4.1.2. Predictive Capability of the Trained ANN Model

**Figure 7:**
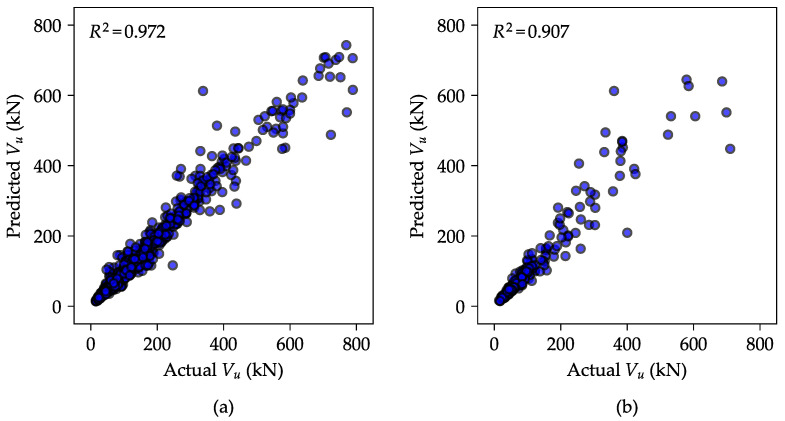
Predictive performance of the ANN model on (**a**) the training set and (**b**) the test set.

Table 3 presents the evaluation metrics of the ANN models trained using the optimized architecture and hyperparameters, assessed on both the training and test sets. A corresponding scatter plot in Figure 7 provides a visual comparison of predicted and actual values. The trained ANN model demonstrated reasonable predictive performance, as it achieved $R^{2}$ values of 0.972 and 0.907 for the training and test sets, respectively. Moreover, the model yielded mean absolute percentage error (MAPE) values of 9.84% and 12.87% on the training and testing sets, respectively.

In addition to the performance metrics, the optimized ANN model yielded COVs of the $V_{\exp}/V_{\mathrm{pred}}$ ratio of 13.31% and 16.91% for the training and test sets, respectively. These values are comparable to, and slightly better than, those reported in Table 1 for a mixed range of beam a/d ratios. This validates the model configuration process employed in this study, including the data normalization procedure and the model optimization guided by Bayesian optimization. These results indicate that the ANN model effectively captures the relationship between the input variables—derived from beam sectional properties and material strength—and the target output (shear strength), while maintaining a reasonable level of generalization to unseen data (test set).

### 4.2. Synthetic Data: Generation via Integration of the GAN Algorithm and ANN Model

After multiple iterations and trials, the CTGANSynthesizer reached its final design, which includes two-layer neural networks with 256 neurons per layer for both discriminator (D) and generator (G). A learning rate of 0.002 and a weight decay rate of 0.0001 are applied to both networks (G and D) during training which uses a batch size of 50. The generator G features an extra embedding dimension of 128. The networks undergo synchronized updates during training to achieve stable convergence.

Following this configuration, the synthesizer was used to generate three datasets of varying sizes—10,000, 100,000, and 1,000,000 instances of independent variables (input features). Each dataset was then fed into the trained ANN model to estimate the ultimate shear strength. The predicted shear strength values, along with their corresponding input parameters, formed three synthetic datasets of different scales.

**Figure 8:**
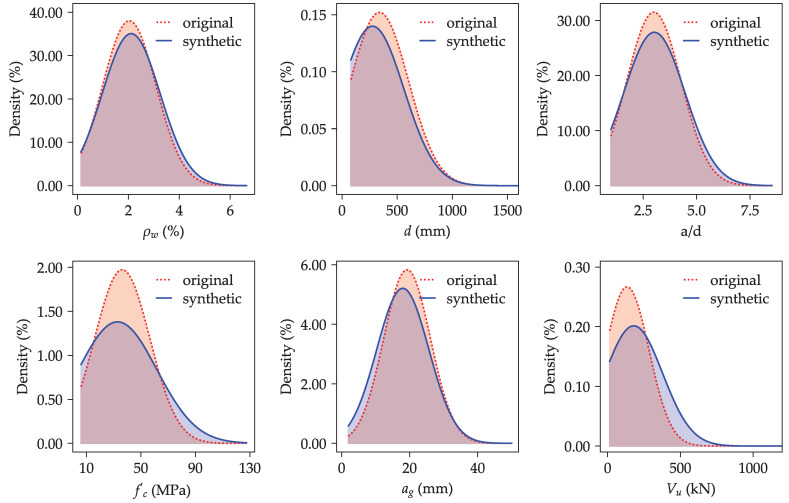
Comparison of the feature distributions of the synthetic data (10,000 instances) and the parent data (original dataset).

Figure 8 presents density plots comparing the distributions of key variables in the original and synthetic datasets, corresponding to the parameters included in the shear strength model in Equation (6). The synthetic data, represented by solid blue curves, exhibit broader distributions across all variables. This broader coverage, particularly at the tails of the distributions, leads to a more uniform representation of the input space. Accordingly, the enriched synthetic dataset provides a robust basis for refining the empirical shear strength model expressed in Equation (6) by capturing a wider range of input scenarios, which could mitigate overfitting to specific data points and enhance the model’s ability to generalize.

### 4.3. Derivation of the Shear Equation: A Data-Centric Bayesian Refinement

Following the procedure outlined in Section 3.5.2, the iterative refinement process was implemented, and the number of iterations in the Bayesian optimization (BO) algorithm was adjusted until convergence was attained. The objective was to maximize the performance of Equation (6) using expanded synthetic datasets of varying sizes—10,000, 100,000, and 1,000,000 instances. Initially, the BO algorithm was executed with $10^{5}$ realizations for each dataset. However, no significant improvement was observed across the three dataset sizes. Accordingly, the smallest dataset (10,000 instances) was selected for further analysis, and the BO algorithm was rerun with $10^{6}$ and $10^{7}$ realizations to investigate the effect of more extensive exploration. Each configuration was repeated twice to ensure the consistency and reliability of the results.

This comprehensive optimization yielded an optimal set of parameters, forming the basis of the proposed unified equation presented in Equation (11) for estimating the shear strength of rectangular RC beams without stirrups. The bounds of each term in the equation were translated into corresponding physical property limits. The equation incorporates both lower and upper bounds for each term, determined through the BO procedure, as detailed below:

(11) $$V_{c}=0.57\cdot \lambda_{d}\cdot {\left(\rho_{w}\right)}^{1/3}\cdot {\left(\frac{a_{g}}{25}\right)}^{1/4}\cdot \lambda_{a}\cdot {\left(f_{c}^{\prime }\right)}^{1/3}\cdot bd$$

$$\mathrm{with}\;a_{g}\geq 25.0\;\mathrm{mm};\;\rho_{w}\leq 6.0\%\;\mathrm{and}\;f_{c}^{\prime }\leq 80.0\;\mathrm{MPa}$$

Therein, the size effect factor $\lambda_{d}$ and shear-span-to-depth ratio factor $\lambda_{a}$ are expressed as

$$1.00\leq \lambda_{d}={\left(\frac{2700}{d}\right)}^{1/3}\leq 2.70\;\mathrm{and}\;0.22\leq \lambda_{a}={\left(\frac{1}{a/d}\right)}^{6/5}\leq 1.00$$

**Figure 9:**
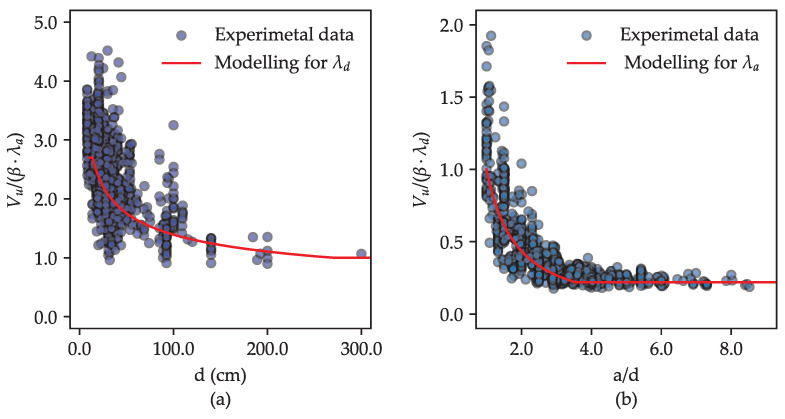
Modeling of (**a**) the size effect $\lambda_{d}$ and (**b**) the shear-span-to-depth ratio effect $\lambda_{a}$ in the proposed equation.

As shown in Equation (11), the proposed shear strength equation differs from the provisions in ACI 318-25 [6] and CSA A23.3-24 [7] by introducing an updated expression for the size effect term ($\lambda_{d}$) and explicitly incorporating the shear-span-to-depth ratio ($\lambda_{a}$). Figure 9 illustrates the resulting models based on the proposed equation, highlighting how the size effect term $\lambda_{d}$ contributes to the total shear strength with varying effective depths (*d*), and how the shear-span-to-depth ratio term $\lambda_{a}$ influences the total shear strength across different a/d ratios. The figure also includes scatter plots of the experimental data for comparison.

As depicted in Figure 9, the forms of $\lambda_{d}$ and $\lambda_{a}$ in the proposed model capture the general trends observed experimentally. Although some extreme data points are not fully matched, the model demonstrates consistent agreement with the overall dataset.

In the plot in Figure 9, the parameter β, defined as

$$\beta =0.57\cdot {\left(\rho_{w}\right)}^{1/3}\cdot {\left(\frac{a_{g}}{25}\right)}^{1/4}\cdot {\left(f_{c}^{\prime }\right)}^{1/3}\cdot bd$$

was introduced specifically to improve the clarity and readability of the plot caption.

In terms of concrete’s compressive strength ($f_{c}^{\prime }$), the maximum limit for $f_{c}^{\prime }$ is 68.9 MPa (implied by $\sqrt{f_{c}^{\prime }}\leq 8.3$) according to ACI 318-25 [6], and 64 MPa (implied by $\sqrt{f_{c}^{\prime }}\leq 8$) according to CSA A23.3-24 [7]. However, the BO algorithm identified a limit for the term ${\left(f_{c}^{\prime }\right)}^{1/3}\leq 4.3$, which corresponds to $f_{c}^{\prime }\leq 80$ MPa. This value is slightly higher than those prescribed by both provisions but closely aligns with the findings of Pendyala et al. [15], who observed that the shear strength of RC beams increases with $f_{c}^{\prime }$ up to approximately 90 MPa. This highlights the capability of BO framework to detect realistic physical limits that are consistent with experimental observations.

It is important to note that existing design codes adopt conservative limits on $f_{c}^{\prime }$ due to limited test data and practical experience with concretes exceeding 70 MPa, as explained in the ACI 318-25 [6] commentary. Furthermore, these codes incorporate safety margins and partial factors that vary across standards to address uncertainties in material behavior, construction quality, and loading conditions. Due to this variability and the safety philosophies embedded in different codes, the present data-driven study does not directly specify or replace code-adopted safety criteria.

### 4.4. Comparative Performance of the Proposed Equation

The proposed equation’s accuracy was evaluated through analysis of a dataset containing 1275 experimental results referenced in Section 3.2. Table 4 compares $V_{\exp}/V_{\mathrm{pred}}$ ratio statistics for the proposed equation against selected provisions: ACI 318-25 [6] and CSA A23.3-24 [7].

The proposed equation yields a mean $V_{\exp}/V_{\mathrm{pred}}$ ratio of 1.110 and a median ratio of 1.085, indicating minimal underestimation relative to the predicted values from ACI 318-25 [6] and CSA A23.3-24 [7]. It also demonstrates superior consistency, evidenced by the lowest standard deviation of 0.243 and a coefficient of variation of 21.87%. Based on an evaluation of 1275 experimental observations, the proposed model provides more consistent predictions and reduced uncertainty across all statistical measures presented in Table 4. These results confirm the improved accuracy and reliability of the proposed equation in predicting shear strength compared to existing design standards.

### 4.5. Parametric Studies

#### 4.5.1. Size Effect of Beam Depth

**Figure 10:**
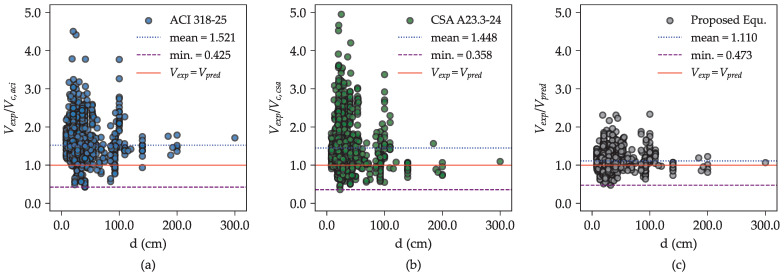
Performance of different shear provisions in predicting the ultimate shear strength of RC beams without stirrups across different beam depths: (**a**) ACI 318-25 [6], (**b**) CSA A23.3-24 [7], and (**c**) the proposed equation.

Figure 10 illustrates the performance of the proposed model by comparing the $V_{\exp}/V_{\mathrm{pred}}$ ratio across various effective beam depths alongside selected shear provisions. For beams deeper than 100 cm, the ACI 318-25 [6] model underestimates shear strength while the CSA A23.3-24 [7] model and the proposed equation generate more accurate predictions, although they slightly overestimate strength.

For beams with depths less than 100 cm, shear strength predictions from all models show bias toward underestimation. Nevertheless, the proposed equation exhibits improved predictive accuracy within this range, despite showing a slight underestimation bias. As shown in Figure 10c, the data points for the proposed equation remain closely aligned with the ideal line $V_{\exp}/V_{\mathrm{pred}}=1$ across varying beam depths. This consistency underscores the effectiveness of incorporating a refined size effect representation into the proposed equation, substantially improving its ability to predict the ultimate shear strength of RC beams without stirrups.

#### 4.5.2. Shear-Span-to-Depth Ratio (a/d)

**Figure 11:**
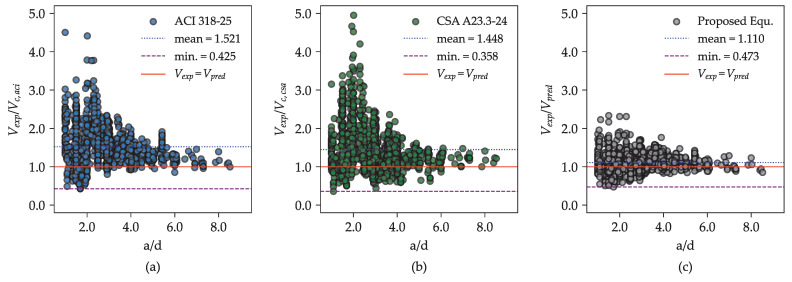
Performance of different shear provisions in predicting the ultimate shear strength of RC beams without stirrups across different a/d ratios: (**a**) ACI 318-25 [6], (**b**) CSA A23.3-24 [7], and (**c**) the proposed equation.

Figure 11 presents the variation of the $V_{\exp}/V_{\mathrm{pred}}$ ratio with different a/d values for the proposed equation and selected design provisions: ACI 318-25 [6] and CSA A23.3-24 [7]. All models exhibit similar trends, generally underestimating shear strength as the a/d ratio decreases. However, the proposed equation shows enhanced predictive accuracy, as indicated by the closer clustering of the $V_{\exp}/V_{\mathrm{pred}}$ values around unity across the full range of a/d ratios. Although it tends to slightly underestimate the strength of beams with a/d ratios below 2.5, the proposed equation still performs marginally better than established provisions such as ACI 318-25 [6] and CSA A23.3-24 [7]. This suggests that the proposed model reliably estimates shear strength for both slender and non-slender RC beams.

From a practical standpoint, the proximity of the $V_{\exp}/V_{\mathrm{pred}}$ ratio to one and the reduced scatter associated with the proposed model imply greater consistency and lower uncertainty. This reliability supports cost-efficient structural designs and contributes to effective risk management by accurately capturing the ultimate shear strength of RC beams without stirrups under varying conditions.

### 4.6. Robustness of Proposed Shear Equation

To evaluate the robustness of the proposed equation, it is tested against experimental results from rectangular RC beams without stirrups using new datasets that are entirely independent of the original data used during model development. These include (1) a dataset compiled by Ahmad and Bhargava [3] for flexural beams with shear-span-to-depth ratios (a/d) ranging from 2.50 to 6.00; (2) a dataset by Chetchotisak et al. [35] for deep beams with a/d ratios between 0.83 and 2.28; and (3) recent test results reported by Daluga et al. [11], covering a/d values from 2.33 to 2.90. The first two datasets were filtered to include only specimens tested after 2007, in order to avoid any overlap with the original dataset used for developing Equation (as detailed in Section 3.2). This filtering ensures a clear separation between development and validation data. The resulting three distinct datasets are summarized in Table 5.

**Figure 12:**
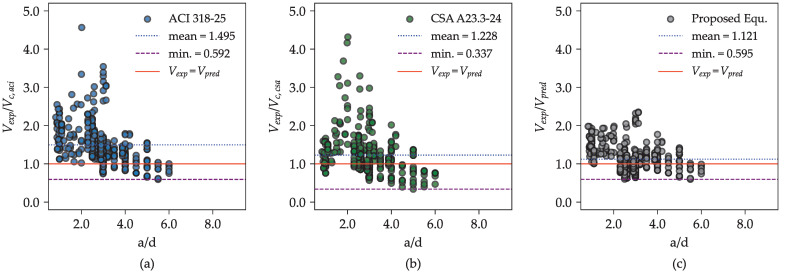
Experimental vs. predicted shear strength ratios for the proposed equation and selected design provisions based on independent datasets (tests conducted from 2007 to 2022): (**a**) ACI 318-25 [6], (**b**) CSA A23.3-24 [7], and (**c**) the proposed equation.

The purpose of this validation is to determine whether the proposed equation maintains consistent and reliable performance when applied to previously unseen experimental data. A statistical summary of the $V_{\exp}/V_{\mathrm{pred}}$ ratios for the proposed equation and selected existing provisions, based on the new datasets, is presented in Table 6 and the scatter plot is illustrated in Figure 12.

As shown in Table 5, for flexural beams (i.e., beams with an a/d ratio between 2.50 and 6.00), the proposed equation yields an average $V_{\exp}/V_{\mathrm{pred}}$ ratio of approximately 1.043. Despite this slight overestimation, it outperforms existing design provisions, including ACI 318-25 [6] and CSA A23.3-24 [7], by providing the closest average ratio to one and the lowest coefficient of variation (COV), reported at 30.49%.

For deep beams (a/d ratio between 0.83 and 2.28), the proposed equation similarly demonstrates superior predictive accuracy compared to both provisions based on the strut-and-tie method. It achieves the closest $V_{\exp}/V_{\mathrm{pred}}$ ratio to one and the lowest COV among the models evaluated. Overall, across the new dataset comprising 375 test results, the proposed equation achieves a mean $V_{\exp}/V_{\mathrm{pred}}$ ratio of 1.121 with a COV of 30.97%. These results confirm the robustness and generalization capability of the proposed model in predicting the shear strength of RC beams without stirrups, even when applied to data beyond the original calibration set.

## 5. Summary and Conclusions

This study presents a novel, data-driven framework for refining the unified shear strength equation for non-prestressed rectangular RC beams without stirrups. By leveraging a comprehensive database of non-prestressed rectangular RC beams without stirrups and integrating advanced computational techniques—namely, artificial neural networks (ANNs), generative adversarial networks (GANs), and Bayesian optimization (BO)—the research develops and validates a predictive model for ultimate shear capacity.

An ANN model was trained on experimental data to predict shear strength with high accuracy. To address the limitation of a relatively small test dataset, 10,000 instances of independent variables (input features) were generated using a GAN algorithm and subsequently input into the trained ANN model to estimate ultimate shear strength. The predicted values, paired with their corresponding input parameters, formed an expanded synthetic dataset—greatly enhancing data coverage and capturing a broader range of design scenarios.

A systematic model development process followed, incorporating key constitutive parameters—including beam depth, longitudinal reinforcement ratio, concrete compressive strength, aggregate size, and the shear-span-to-depth ratio—to express shear stress ($V_{c}/bd$) using power-law relationships. The BO framework was employed to fine-tune the model coefficients, efficiently exploring the parameter space through $10^{5}$, $10^{6}$, and $10^{7}$ realizations. This exhaustive search ensured convergence and enabled the identification of statistically significant features.

The final outcome is a robust shear strength equation—simple in form yet highly accurate. Built upon physical principles and refined through data-driven modeling, the proposed model provides consistent and reliable predictions with significantly reduced uncertainty compared to existing provisions such as ACI 318-25 and CSA A23.3-24. Its performance has been benchmarked against experimental tests conducted over more than 60 years (1958–2022).

A key contribution of this research is the formulation of a unified equation that reasonably predicts the shear strength of both shear-slender and non-shear-slender beams, without relying on restrictive assumptions such as specific shear-span-to-depth (a/d) thresholds. The proposed model outperforms traditional methods in terms of prediction accuracy and uncertainty reduction and provides a versatile and effective solution across a wide range of structural scenarios.

## Figures and Tables

**Table 1:** Recent studies on data-driven modeling of shear strength of RC beams without stirrups.

Reference	Data Size	a/d Ratio	Optimal Algorithm	Mean * (COV)	Key Findings
Lee and Kang [18]	1149 instances	2.50–15.0	GPR ($R^{2}=0.940$)	1.00 (4.7%)	Outperforms existing shear provisions (ACI 318, AASHTO), showing superior accuracy.
Prayoonwet et al. [19]	Dataset A: 1312 Dataset B: 719 Dataset C: 285	0.30–7.29 0.27–2.50 0.50–4.00	Neural network	0.97 (27.1%) 0.98 (17.2%) 1.00 (51.4%)	Achieves significantly higher prediction accuracy than traditional models (NZS, CSA, ACI, JSCE).
Megahed [23]	840 instances	0.27–2.50	CatBoost ($R^{2}=0.947$)	1.01 (8.5%)	Demonstrates superior predictive capability over standard methods (ACI 318, EC2).
David et al. [24]	784 instances	2.40–8.10	XGBoost ($R^{2}=0.974$)	1.00 (14.0%)	Achieves the lowest error margin, surpassing mechanics-based models (Tran’s NLT model, multi-action shear model, compression chord model).

* Mean and coefficient of variation (COV) of the $V_{\exp}/V_{\mathrm{pred}}$ ratio.

**Table 2:** Range of independent variables (predictor) and their corresponding ultimate shear strength (dependent variable or target) within the dataset.

Variable	Min. Value	Mean Value	Max. Value	Std. Dev.	Type
X1: b (mm)	100.00	241.02	3000.00	237.25	input
X2: h (mm)	100.00	386.13	3140.00	278.60	input
X3: d (mm)	80.00	339.56	3000.00	262.04	input
X4: bearing * (mm)	10.00	104.95	600.00	78.41	input
X5: $\rho_{w}$ (%)	0.14	2.03	6.64	1.05	input
X6: $a_{g}$ (mm)	2.00	19.23	50.00	6.84	input
X7: a/d (–)	1.00	3.00	8.52	1.27	input
X8: $f_{y}$ (MPa)	267.00	462.82	1779.00	146.48	input
X9: $f_{c}^{\prime }$ (MPa)	6.10	36.31	127.50	20.22	input
y: $V_{u}$ (kN)	14.50	134.39	1575.00	149.49	output

* Bearing: the width of the steel loading plate used during experiments.

**Table 3:** Summary of four evaluation metrics for assessing the predictive capability of the ANN model on the training and test sets.

Dataset	Performance Metrics				$V_{\exp}/V_{\mathrm{pred}}$ Ratio	
	*R* ^2^	MAE (kN)	RMSE (kN)	MAPE (%)	Mean	COV (%)
Training set: 1020 instances	0.972	12.72	25.91	9.84	1.050	13.41
Test set: 255 instances	0.907	18.44	38.67	12.87	1.041	16.91

**Table 4:** Summary of statistical parameters comparing the proposed equation with various provisions for predicting the ultimate shear strength of RC beams without stirrups.

Model	$V_{\exp.}/V_{\mathrm{pred}.}$ (1275 Instances)				Performance	
	Mean	Median	Stdev.	COV * (%)	MAE (kN)	MAPE (%)
ACI 318-25 [6]	1.521	1.432	0.475	31.24	51.28	32.37
CSA A23.3-24 [7]	1.448	1.312	0.594	40.99	44.75	29.63
Proposed Equation	1.110	1.085	0.243	21.87	27.23	17.00

* COV: Coefficient of Variation.

**Table 5:** Overview of new experimental datasets used in robustness evaluation.

References	Dataset	b (mm)	d (mm)	a/d	$\rho_{w}$ (%)	$f_{c}^{\prime }$ (MPa)	$V_{u}$ (kN)
Ahmad and Bhargava [3]	275	100.0–613.0	100.0–1251.0	2.5–6.0	0.33–4.76	23.20–194	14.7–882.3
Chetchotisak et al. [35]	70	80.0–400.0	180.0–1440.0	0.83–2.28	0.69–3.62	18.0–87.0	85.0–1544.0
Daluga et al. [11]	30	229.0–610.0	267.0–1067.0	2.3–2.9	0.63–0.98	18.62–34.47	48.9–485.9

**Table 6:** Comparison of proposed equation with other shear provisions with respect to $V_{\exp}/V_{\mathrm{pred}}$ using new experimental data.

References	Dataset	a/d	ACI 318-25 [6]		CSA A23.3-24 [7]		Proposed Equation	
			Mean	COV (%)	Mean	COV (%)	Mean	COV (%)
Ahmad and Bhargava [3]	275	2.50–6.00	1.401	33.64	1.147	36.47	1.043	30.49
Chetchotisak et al. [35]	70	0.83–2.28	1.840	31.17	1.619	46.12	1.497	17.21
Daluga et al. [11]	30	2.33–2.90	1.553	20.40	1.058	18.32	0.961	13.66
Overall	375	0.83–6.00	1.495	34.12	1.228	42.30	1.121	30.97

## Data Availability

The original contributions presented in this study are included in the article. Further inquiries can be directed to the corresponding author.

## Abbreviations

*a*	shear span length, mm
$a_{g}$	maximum aggregate size in concrete composition, mm
*b*	width of beam, mm
*d*	effective depth of beam, mm
$f_{ck}$	specified compressive strength of concrete, MPa
$V_{c}$	ultimate shear strength of RC beam without shear reinforcement, kN
$\lambda_{a}$	shear-span-to-depth ratio (a/d) effect factor
$\lambda_{d}$	size effect factor related to effective depth of beam
$\rho_{w}$	longitudinal tensile reinforcement steel ratio, %
